# Engineering Fe-Modified Zeolitic Imidazolate Frameworks (Fe-ZIF-8 and Fe-ZIF-67) via In Situ Thermal Synthesis for Enhanced Adsorption of Malachite Green from Aqueous Solutions: A Comprehensive Study of Isotherms, Kinetics, and Thermodynamics

**DOI:** 10.3390/nano15141097

**Published:** 2025-07-15

**Authors:** Alireza Pourvahabi Anbari, Shima Rahmdel Delcheh, Muhammad Kashif, Alireza Ranjbari, Mohammad Karbalaei Akbari, Serge Zhuiykov, Philippe M. Heynderickx, Francis Verpoort

**Affiliations:** 1 Center for Green Chemistry and Environmental Biotechnology, Ghent University Global Campus, Incheon 406-840, Republic of Korea; alireza.pourvahabi@ghent.ac.kr (A.P.A.); m.kashif@ghent.ac.kr (M.K.); alireza.ranjbari@ugent.be (A.R.); mohammad.akbari@ugent.be (M.K.A.); serge.zhuiykov@ghent.ac.kr (S.Z.); philippe.heynderickx@ghent.ac.kr (P.M.H.); 2 Department of Chemistry, Faculty of Science, Ghent University, 9000 Gent, Belgium; 3 State Key Laboratory of Advanced Technology for Materials Synthesis and Processing, Wuhan University of Technology, 430070 Wuhan, China; shima.rahmdel@yahoo.com; 4 Department of Green Chemistry and Technology, Faculty of Bioscience Engineering, Ghent University, 9000 Ghent, Belgium; 5 Department of Solid-State Sciences, Faculty of Science, Ghent University, 9000 Ghent, Belgium; 6 Research School of Chemical and Biomedical Technologies, National Research Tomsk Polytechnic University, Lenin Avenue 30, 634050 Tomsk, Russia; 7 Joint Institute of Chemical Research (FFMiEN), Peoples Friendship University of Russia (RUDN University), 117198 Moscow, Russia

**Keywords:** organic dyes, malachite green, adsorption, zeolitic imidazole framework (ZIF), nanocomposites

## Abstract

Given the adverse effects of organic dyes from aqueous solutions on human physiology and the ecological system, establishing an effective system for their elimination is imperative. This study employs the in situ thermal (IST) method to synthesize nanocomposites comprising zeolitic imidazole frameworks, specifically Fe-ZIF-8 and Fe-ZIF-67. The investigation offers a comprehensive evaluation of the properties of these nano-adsorbents for the removal of malachite green (MG). The results indicate a significantly increased adsorption capacity of up to 495 and 552 mg g^−1^ for Fe-ZIF-8 and Fe-ZIF-67, respectively. Furthermore, they demonstrate removal efficiencies of up to 90% and 95% for MG, respectively. Parameters associated with the adsorption process are derived from isotherms and removal kinetics, specifically the Freundlich model and the pseudo-second-order kinetics model, respectively. The enhanced adsorption capacity observed in Fe-ZIF-8 and Fe-ZIF-67 can be attributed to π–π stacking interactions, hydrogen bonding, and electrostatic attraction. After undergoing three cycles, both adsorbents consistently exhibit a high removal efficiency of approximately 85%, indicating notable structural integrity and outstanding potential for repeated use. The examined adsorbents display exceptional efficacy, favorable stability, and substantial specific surface area, underscoring their remarkable adsorption capabilities. The nanocomposites comprising Fe-ZIF-8 and Fe-ZIF-67 demonstrate considerable potential as highly favorable options for the elimination of MG and other cationic organic dyes from aqueous environments.

## 1. Introduction

Rapid industrialization has brought notable advancements but also severe environmental challenges, including water pollution from a variety of contaminants such as dyes, heavy metals, pharmaceuticals, and pesticides. Among them, synthetic dyes pose a major threat to aquatic systems due to their toxicity, persistence, and resistance to conventional treatment methods [1]. Malachite green (MG), a triphenylmethane dye widely used in the textile and aquaculture industries, is of particular concern because of its long-term environmental persistence and potential carcinogenic and mutagenic effects [2].

Various treatment technologies have been developed to remove MG from wastewater, including oxidation [3], membrane filtration [4], ozonation [5], and chlorination [6]. However, these techniques often suffer from limitations such as high cost, secondary pollution, or incomplete removal. Adsorption has emerged as one of the most promising methods due to its simplicity, efficiency, reusability, and low cost [7,8,9]. Despite significant progress, conventional adsorbents such as activated carbon, zeolites, graphene oxide, and biopolymers often struggle with low adsorption capacity, limited stability, and poor recyclability [10].

Recently, metal–organic frameworks (MOFs), particularly zeolitic imidazolate frameworks (ZIFs), have gained attention as advanced adsorbents owing to their high surface area, tunable porosity, and exceptional thermal and chemical stability [11]. ZIF-67, composed of Co^2+^ and 2-methylimidazole, shows promise in dye adsorption due to its porosity and potential for redox-active cobalt sites [12]. Nevertheless, traditional synthesis methods of ZIFs are time-consuming and solvent-intensive. Additionally, powdered ZIFs pose challenges in separation after adsorption, prompting interest in modified and magnetic adsorbents for improved recovery [13]. Incorporation of higher valent metal ions into ZIFs creates coordinatively unsaturated sites and framework defects, which enhance guest accessibility and provide new adsorption modes beyond pure physisorption. Fe(III), with its high charge density and strong coordination affinity for nitrogen donors, was chosen to induce both electrostatic/coordination interactions with cationic dyes and magnetic properties to aid separation. Recent work has shown that the deliberate introduction of a small fraction of paramagnetic centers into MOF linkages can yield intrinsic room temperature ferromagnetism in 2D semiconducting frameworks, confirming the feasibility of tuning magnetic behavior via dopant engineering [14].

In this work, we report the synthesis of highly porous Fe-ZIF-8 and Fe-ZIF-67 nanocomposites using an in situ thermal (IST) method, a solvent-free, green synthesis strategy that eliminates the need for external heating, additives, or complex post-treatment. The prepared adsorbents were characterized by XRD, FTIR, BET, TEM, TGA, FE-SEM, and other techniques. Their efficiency for MG removal was studied under various parameters (pH, contact time, dosage, and temperature), and adsorption performance was evaluated using kinetic and isotherm models. This study aims to demonstrate the effectiveness of IST-synthesized Fe-ZIFs as sustainable and high-performance materials for water purification.

## 2. Materials and Methods

All chemicals used in the experiment were of analytical grade, and no further purification procedures were conducted on them. The chemicals used include cobalt (II) acetylacetonate (≥99%, Aldrich, Co(C_5_H_7_O_2_)_2_, Product code: 14024-48-7), zinc acetylacetonate hydrate (99%, Aldrich, Zn(C_5_H_7_O_2_)_2_·xH_2_O, Product code: 108503-47-5), iron (III) acetylacetonate (99%, Aldrich, Fe(C_5_H_7_O_2_)_3_, Product code: 14024-18-1), 2-methylimidazole (≥99%, Aldrich, C_4_H_6_N_2_, Product code: 693-98-1), sodium hydroxide (NaOH, Aldrich, product code: 1.06462.1000), hydrogen chloride (≥99%, HCl, Aldrich, product code: 1.09057.5000), and malachite green (≥99%, Aldrich, C_23_H_25_N_2_, Product code: 2437-29-8). The pH of the solutions was set using 0.1 M hydrochloric acid (≥99%, Aldrich, product code: 1.09057.5000) and 0.1 M sodium hydroxide (≥99%, Aldrich, product code: 1.06498.5000).

### 2.1. Synthesis of Fe-ZIF-8 and Fe-ZIF-67

The Fe-ZIF-67 compound was synthesized by combining 2-methylimidazole (246 mg), Co(II) acetylacetonate (231 mg), and Fe(III) acetylacetonate (35 mg). The blend was manually prepared in a mortar at room temperature under normal atmospheric pressure for an interval of 5 min. The Fe-ZIF-8 synthesis was performed with a methodology akin to that employed for the cobalt source, wherein the substitution of zinc acetylacetonate hydrate (236 mg) was utilized. The experimental procedure involved the creation of a solution by mixing 246 mg of 2-methylimidazole with 36 mg of Fe(III) acetylacetonate. Subsequently, the powder combination acquired was meticulously placed into an alumina boat with dimensions of 18 × 80 × 12 mm^3^ and subsequently inserted into an alumina tube with an outer diameter of 60 mm and a length of 1000 mm. The complete apparatus was subsequently positioned inside a muffle furnace (model SH 1500, manufactured by SAMHEUNG (Seoul) in the Republic of Korea). The synthesis technique was conducted under regulated conditions in a controlled environment, utilizing an inert atmosphere. A nitrogen stream was employed at a flow rate of 100 cm^3^·min^−1^. The experimental methodology encompassed the utilization of a two-step temperature program. The temperature was incrementally raised from the initial ambient room temperature to 100 °C over 30 min. Following that, the temperature was then augmented from 100 °C to 200 °C at a consistent rate of 5 °C per minute. Upon attaining the maximum temperature, it was maintained for one hour. After the completion of the cooling procedure, the resulting zeolitic imidazolate frameworks (ZIFs) were collected.

### 2.2. Characterization Instruments

X-ray diffraction (XRD) was employed to explore the organization and composition of substances, including chemical bonding, crystal formations, and phase transitions. XRD analyses were performed utilizing a 1-dimensional high-speed detector called the D/teX Ultra Silicon Strip Detector, which was developed by Rigaku (Tokyo, Japan). The analysis was conducted over the angular range of 5° to 70°, employing a scanning rate of 2°/min. The X-ray photoelectron spectroscopy (XPS) analysis was conducted using a Thermo Fisher Scientific, (Leicestershire, UK) K-alpha XPS (mono) instrument. The utilization of Fourier-transform infrared spectroscopy (FT-IR) was employed in order to carry out functional group analysis on the materials. The equipment employed for this study was the Nicolet Avatar 360, which was produced in the United States. To examine the morphological attributes, the investigators utilized field emission scanning electron microscopy at a voltage of 30 kV, achieving a resolution of 1.2 nm. The Field Emission Scanning Electron Microscopes (FE-SEM) equipment applied in this study was produced by JEOL (Tokyo, Japan). The qualitative and quantitative examination of the elemental composition was performed using an Inductively Coupled Plasma Optical Emission Spectrometer (ICP-OES) manufactured by Agilent in Santa Clara, CA, USA. The application of energy dispersive X-ray analysis (EDX) was employed to determine the elemental composition and molar ratios of the adsorbents. The study aimed to analyze the various attributes of porosity, including size distributions, pore volume, average pore size, pore classification, and specific surface areas. To do this, the research utilized the Brunauer–Emmett–Teller (BET) method, employing an Autosorb IQ instrument produced by Quantachrome (Graz, Austria). To obtain further insights into thermal stability and mass variations, the investigators utilized thermogravimetric analysis (TGA, Q50, USA) with a heating rate of 5 °C/min, under an N_2_ atmosphere. The UV-VIS spectrophotometer produced by Optizen in Daejeon, Republic of Korea was utilized to determine the concentration of the MG, at λ_max_ 619 nm.

### 2.3. Adsorption Experiments

The adsorption experiments were carried out with a water bath shaker manufactured by Daihan (Deajeon, Republic of Korea). In each experimental trial, a standardized amount of 17.5 milligrams of adsorbents was carefully put into a dark Erlenmeyer flask to minimize the potential effects of light. The flask was filled with a 100 mL volume of a solution of MG. The pH of the solution was altered by the introduction of a 0.1 M HCl or NaOH solution. The value of the pH was measured using a pH meter (Toledo, Columbus (OH), USA). The flask was subjected to agitation at a rotational speed of 200 revolutions per minute (rpm) under normal environmental conditions for 2 h. Following that, the solution was subjected to centrifugation at a rotational speed of 5000 revolutions per minute for 3 min. Subsequently, the substance underwent filtration utilizing a sterile syringe filter made of polyethersulfone (PES) material, with a particle size of 0.2 µm. The concentration of Malachite Green was obtained from UV-VIS measurements at a specific wavelength of 619 nm, using a UV-VIS spectrophotometer (Optizen, Optizen pop, Republic of Korea). Equations (1) and (2) can be employed to determine the adsorption capacity and removal efficiency, respectively, of the adsorbents.

(1) $$q_{t}=\frac{\left(C_{0}-C_{t}\right)V}{m}$$

(2) $$w\left(\%\right)=\frac{\left(C_{0}-C_{t}\right)}{C_{0}}\times 100$$

where q_t_ (mg/g), w (%), C_0_ (mg/L), C_t_ (mg/L), V (L), m (mg) are the adsorption capacity, the removal efficiency, the initial concentration, solution concentration, adsorption time, solution volume and the mass of adsorbent, respectively.

### 2.4. Adsorption Isotherms

The adsorptive capacity of the materials was evaluated using equilibrium isotherm models [15]. All isotherm experiments were conducted at a controlled pH of 6 (adjusted with 0.1 M HCl/NaOH), consistent with the kinetic studies. A quantity of 17.5 mg of each adsorbent (Fe-ZIF-8 and Fe-ZIF-67) was added to 50 mL MG solutions of varying initial concentration (20–100 mg·L^−1^). The suspensions were stirred at 200 rpm and maintained at the target temperature (25 °C, 35 °C, or 45 °C) until equilibrium (~120 min) was reached. Duplicate experiments were performed, and residual MG concentrations were measured to calculate q_e_.

### 2.5. Langmuir Isotherm

The Langmuir adsorption isotherm is a prominent theoretical model that assists in understanding the adsorption capacity and equilibrium behavior of an adsorbent. This model presupposes the presence of a completely saturated monolayer of adsorbed chemicals on the adsorbent surface. Additionally, it assumes that there exists a predetermined number of active sites that are available for the process of adsorption. Furthermore, this model assumes that the adsorption and desorption energies are constant and unvarying at a specific temperature, and lateral interactions are not considered [16,17]. The mathematical expression for the Langmuir isotherm is denoted as follows:

(3) $$\frac{C_{e}}{q_{e}}=\frac{1}{q_{m}\;K_{L}}+\frac{C_{e}}{q_{m}}$$

describing the relationship between the liquid MG equilibrium concentration C_e_ (mg/L), the adsorption capacity q_e_ (mg/g), the maximum adsorption capacity on the monolayer surface of the adsorbent q_m_ (mg/g), and the Langmuir isotherm constant K_L_ (L/mg).

### 2.6. Freundlich Isotherm

The Freundlich adsorption isotherm is relevant in the range of low to moderate concentrations, and it comes with a non-ideal behavior of the adsorbent surface, i.e., the surface is heterogeneous and adsorption energy can be different depending on the specific site and fractional coverage. The Freundlich isotherm is capable of accommodating various types of interactions with ions that are observed in aqueous solutions [11,18]. It is mathematically represented in a linear form as follows:

(4) $$q_{e}=K_{f}\;C_{e}^{\frac{1}{n}}$$

where q_e_ (mg/g) is the adsorption capacity at equilibrium, K_f_ (mg/g) is the Freundlich coefficient, representing the adsorption capacity relative to the adsorbate concentration, C_e_ (mg/L) is the equilibrium adsorbate concentration in the solution, and n is the adsorption intensity parameter. It is related to the favorability of adsorption. For instance, if 1/n = 0, the isotherm is considered irreversible. If 0 < 1/n < 1, the isotherm is desirable. Conversely, if 1/n > 1, the isotherm is unfavorable. Furthermore, the value of n provides insights into the nature of the adsorption. If n < 1, it signifies chemisorption, below monolayer coverage. The case n = 1 corresponds to a linear adsorption process, and the case n > 1 hints towards physical adsorption [19].

### 2.7. Temkin Isotherm

This model is formulated based on the premise that the adsorption heat for each species inside the adsorbed layer experiences a linear decline with increasing surface coverage. This phenomenon exhibits a contrasting pattern to a logarithmic reduction, which is a result of the intermolecular contacts between the adsorbate molecules. Moreover, the existing theoretical framework proposes the existence of a homogeneous distribution of binding energies throughout the adsorbent’s surface [20,21]. The mathematical representation of the Temkin isotherm model is a linear equation.

(5) $$q_{e}=B\ln A_{T}+B\ln C_{e}$$

The variable q_e_ (J/mol) denotes the equilibrium adsorption capacity, whereas B (J/mol) represents the constant of the Temkin isotherm, which characterizes the adsorption heat. A_T_ (L/g) is the Temkin constant, which is determined by plotting q_e_ against ln (C_e_) in a linear manner. The Temkin isotherm model offers insights into the adsorption phenomenon by examining the relationship between the heat of adsorption and the variation in surface coverage.

### 2.8. Dubinin–Radushkevich (D-R) Isotherm Model

The Dubinin–Radushkevich (D-R) isotherm model is a theoretical model used to describe the adsorption of gas molecules onto solid surfaces. It is an extension of the Langmuir isotherm and is often applied to describe adsorption on heterogeneous surfaces with varying adsorption energies. The D-R isotherm equation is as follows:

(6) $$q=Q_{exp}(-\beta {\;\varepsilon }^{2})$$

where q adsorption capacity, Q_exp_ is the theoretical monolayer capacity of the adsorbent, β is a constant related to the adsorption energy, ε is the Polanyi potential. The Dubinin–Radushkevich isotherm model is often used for physical adsorption, where adsorbate molecules are held to the adsorbent surface by weak van der Waals forces. The model assumes a Gaussian energy distribution of adsorption sites on the surface.

### 2.9. Kinetic Study

To ascertain a suitable depiction of the adsorption rate and gain a more comprehensive understanding of the underlying mechanisms encompassing chemical reactions, diffusion control, and mass transmission in the adsorption procedure [22], the following kinetic models were employed: the pseudo-first-order, pseudo-second-order, and Elovich models. The examination of the kinetics of adsorption plays a pivotal role in determining the best conditions for batch adsorption processes on a significant scale [23,24]. The kinetics of the adsorption procedure were examined using three different kinetic models: the pseudo-first-order, pseudo-second-order, and Elovich kinetic models. To determine the kinetic coefficient, the removal of MG was studied under the optimal experimental conditions of dosage = 17.5 mg, temperature = 25 °C, initial concentration = 80 mg/L, pH = 6, and under adsorption time of 15 to 420 min.

### 2.10. Pseudo-First-Order Kinetic Model

The pseudo-first-order kinetic model is commonly utilized among other kinetic models and is regarded as a favorable option for estimating the adsorption rate. The fundamental premise of this model is predicated on the existence of a direct correlation between the rate at which the sorbate fills binding sites and the number of available empty sites [25]. The pseudo-first-order kinetic model may be defined in the following manner:

(7) $$\log(q_{e}-q_{t})=logq_{e}-(\frac{k_{1}t}{2.303})$$

The variable k_1_ (min^−1^) in the given equation denotes the pseudo-first-order rate constant. The values of k_1_ and q_e_ are derived from the slope and intercept, respectively, of the linear graph depicting the logarithm of the difference between q_e_ and q_t_ as a function of time [26].

### 2.11. Pseudo-Second-Order Kinetic Model

The pseudo-second-order kinetic model is based on the underlying assumption that the rate at which sorbate molecules are adsorbed into binding sites is precisely proportional to the square root of the number of empty sites [27]. This model suggests that chemisorption significantly impacts the rate and mechanism of adsorption. The mathematical representation of the pseudo-second-order kinetic model is stated as follows:

(8) $$\frac{t}{q_{t}}=\frac{1}{k_{2}q_{e}^{2}}+\frac{t}{q_{e}}$$

The variable k_2_ (expressed in units of g mg^−1^ min^−1^) denotes the adsorption rate constant in the pseudo-second-order model equation. The values of k_2_ and q_e_ are derived from the slope and intercept, respectively, of the linear graph depicting t/q_e_ against t.

### 2.12. Elovich Kinetic Model

The Elovich equation is a kinetic model used to describe activated chemical adsorption [28]. It is expressed as follows:

(9) $$q_{t}=\frac{1}{\beta }\ln\left(\alpha \beta \right)+\frac{1}{\beta }\ln t$$

Here, α (mg min/g) represents the initial adsorption rate constant, while β (g/mg) is related to both the degree of surface coverage and the activation energy for chemisorption. These parameters, α and β, can be determined from the linear plot of q_t_ versus ln t. The Elovich model offers valuable insights into the kinetics of adsorption and the underlying processes that occur during the adsorption of a solute onto the surface of the adsorbent [29].

### 2.13. Analysis of Thermodynamic

The thermodynamic properties, specifically the alterations in standard Gibbs free energy (ΔG°), standard enthalpy (ΔH°), and standard entropy (ΔS°), were computed utilizing the provided thermodynamic equations. The objective of these calculations was to assess the feasibility of the adsorption processes and offer insights into the inherent energy conversions that take place [30].

Equation (9) is utilized for the determination of the distribution adsorption coefficient (K_D_).

(10) $$K_{D}=\frac{q_{e}}{C_{e}}$$

Here, q_e_ represents the equilibrium adsorption capacity (mg/g), and C_e_ is the equilibrium concentration of malachite green in the solution (mg/L) [31]. The standard free energy change (ΔG°, J/mol), standard enthalpy (ΔH°, kJ/mol), and standard entropy (ΔS°, J/mol·K) can be determined from Equations (10) and (11).

Δ*G*° = −*RT ln*(*K_D_*) (11)

(12) $$\ln\left(K_{D}\right)=\frac{{∆S}^{{}^{\circ}}}{R}-\frac{{∆H}^{{}^{\circ}}}{RT}$$

where R is the universal gas constant (8.314 J/mol·K).

The values of ΔH° (standard enthalpy) and ΔS° (standard entropy) can be obtained by analyzing the slope and intercept of the graph depicting the natural logarithm of K_D_ (ln (K_D_)) as a function of the reciprocal of temperature (1/T). In general, positive values for ΔH° (standard enthalpy) and ΔG° (standard Gibbs free energy) denote that the adsorption processes are endothermic and non-spontaneous. Conversely, in cases when ΔH° and ΔG° display negative values, it can be inferred that the adsorption phenomena are exothermic and they spontaneously take place. The presence of negative values for ΔS° corresponds to a decrease in degrees of freedom of the adsorbate. The categorization of the adsorption process can be determined based on the magnitude of ΔH°. Physisorption is observed under conditions where the change in enthalpy (ΔH°) falls within the range of 0 to 20 kJ/mol or the change in Gibbs free energy (ΔG°) falls within the range of −20 to 0 kJ/mol. On the other hand, chemisorption occurs when the change in enthalpy (ΔH°) is between 80 and 400 kJ/mol or the change in Gibbs free energy (ΔG°) is between −400 and −80 kJ/mol [32,33].

## 3. Results and Discussions

### 3.1. Characterization

**Figure 1:**
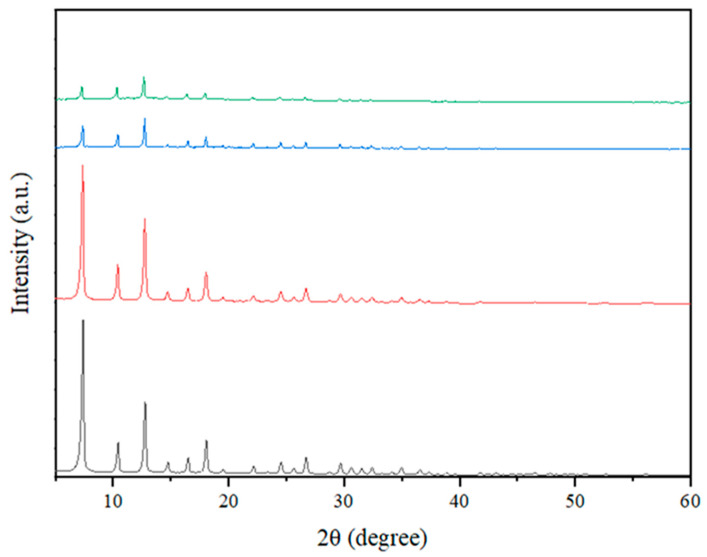
XRD patterns of samples and Fe-ZIF-67 produced using the IST method. (**―**) Fe-ZIF-67, Simulated ZIF-67 (**―**), Fe-ZIF-8 (**―**), and Simulated ZIF-8 (**―**).

The XRD patterns of Fe-ZIF-8 and Fe-ZIF-67, synthesized using the IST approach, are shown in Figure 1. The experimental results showed that the crystalline structure of all ZIFs exhibited a significant degree of order. These patterns exhibit a robust link with prior research findings. The XRD spectrum has distinct peaks at precise angles, specifically 29.7°, 24.6°, 22.3°, 18.2°, 16.8°, 14.7°, 12.9°, 10.6°, and 7.4°. The angles listed in a sequential manner correspond to the diffraction planes (004), (233), (114), (222), (013), (022), (112), (002), and (011) [34]. The peaks observed in the Fe-ZIF-67 pattern closely resembled those found in the pure ZIF-67, indicating the successful synthesis of Fe-ZIF-67. The presence of iron in the sample may have contributed to the somewhat reduced intensities found for the (013) and (011) peaks, as compared to ZIF-67 [35]. Nevertheless, notable disparities were identified in the 2θ angles and peak intensities of the doped materials. The effect seen in this study can be ascribed to the alteration of ZIF-8′s crystal structure due to the substitution of Zn^2+^ ions with Co^2+^ or Fe^3+^ ions [36]. The XRD patterns acquired for Fe-ZIF-8 and Fe-ZIF-67 demonstrate that the introduction of a small quantity of iron did not result in notable alterations to the ZIF crystalline architecture. Moreover, following the introduction of iron, there was a marginal reduction in the magnitude of the (112) and (222) diffraction peaks, indicating the possible replacement of cobalt (Co) or zinc (Zn) atoms with iron, as suggested by reference [37].

**Figure 2:**
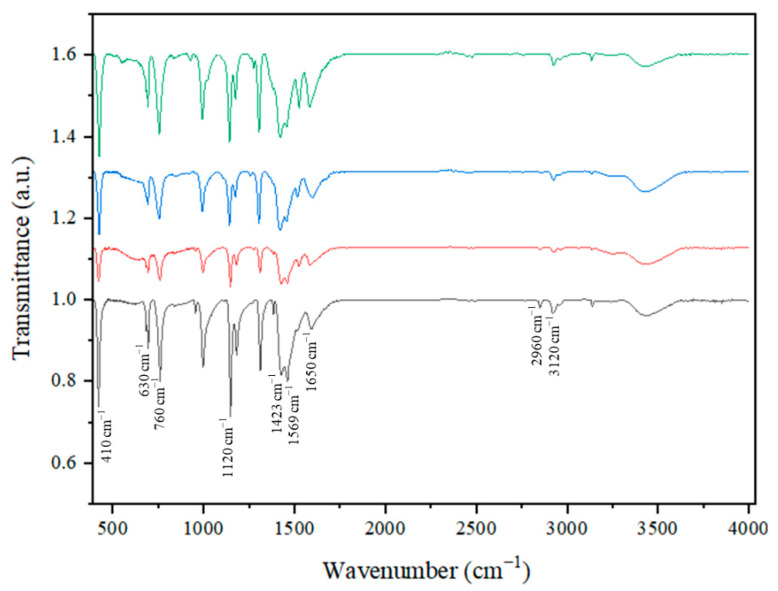
FT-IR spectra of samples produced using the IST method. (**―**) Fe-ZIF-67, ZIF-67 (**―**), Fe-ZIF-8 (**―**), and ZIF-8 (**―**).

Figure 2 depicts the application of infrared spectroscopy in the identification of functional groups in the nanocomposites of the adsorbents. The process of synthesizing Fe-ZIF nanocomposites was carried out utilizing 2-methylimidazole as the organic linker. Distinct stretching and bending patterns characteristic of 2-methylimidazole were seen in the absorbance spectra within the area of 710–1540 cm^−1^ [38]. The presence of absorption bands at a wavenumber of 760 cm^−1^ implies the occurrence of out-of-plane bending, whereas the wavenumber range of 1050–1450 cm^−1^ signifies the bending motion taking place within the same plane of the carbon–nitrogen (C-N) bond in 2-methylimidazole. Furthermore, the observed absorption bands at wavenumbers 3120 and 2960 cm^−1^ were allocated to the aromatic and aliphatic C-H stretching vibrations of the 2-methylimidazole molecule, respectively [39]. The spectral region spanning from 1250 to 1550 cm^−1^ exhibits a conspicuous peak, which can be ascribed to the vibrational modes linked to the bending and stretching of the imidazole ring. Concurrently, the existence of peaks at wavenumbers 410 and 1650 cm^−1^ might be attributed to the stretching vibrations of Zn-N and C-N bonds, correspondingly, as supported by previous studies [40]. The spectra displayed two distinct peaks: the initial peak, located at a wavenumber of 1569 cm^−1^, refers to the stretching vibration of the C=N bond, whilst the second peak, recorded at 1423 cm^−1^, pertains to the stretching vibration of the C-H bond within the CH_2_ area. It is important to acknowledge that the occurrence of Co-N bonds was directly correlated with a distinct peak detected at 1120 cm^−1^ [40]. Additionally, the stretching mode of the Fe–N bond is detected across the frequency spectrum of 630 cm^−1^ in all samples within the series. The discovery cited above indicates the potential formation of complexes involving Fe^3+^ and 2-methylimidazole, resulting in the formation of coordination bonds within the metal–organic framework [41]. The results confirm that the structural integrity of zeolitic imidazolate frameworks (ZIFs) is unchanged following the inclusion of bimetallic compounds. Moreover, it can be inferred that the reduction in the intensities of the absorption bands associated with the incorporation of metal, as compared to the original state of the ZIFs, can be ascribed to the existence of iron (Fe) particles within the framework [42].

**Figure 3:**
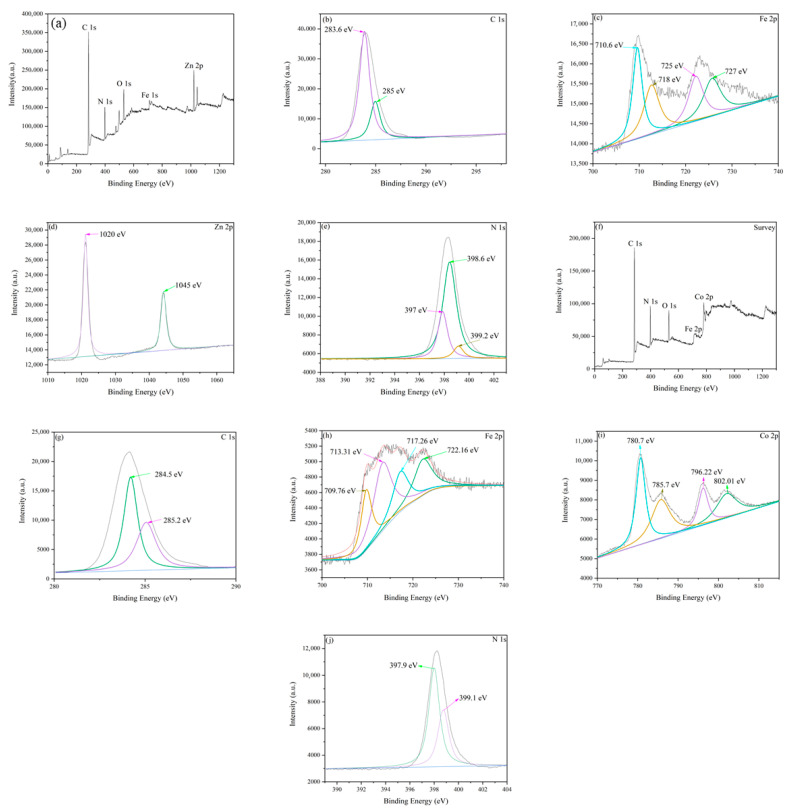
XPS spectra of Fe-ZIF-8 and Fe-ZIF-67 produced using the IST method. (**a**) Survey spectrum of Fe-ZIF-8, with elemental compositions: C 1s (**b**), Fe 2p (**c**), Zn 2p (**d**), and N 1s (**e**). (**f**) Survey spectrum of Fe-ZIF-67, with elemental compositions: C 1s (**g**), Fe 2p (**h**), Zn 2p (**i**), and N 1s (**j**).

The surface chemistry of Fe-ZIF-8 and Fe-ZIF-67 was analyzed using X-ray photoelectron spectroscopy (XPS), as shown in Figure 3a,f. Figure 3a presents the survey spectra of these ZIFs, confirming the presence of elements such as Zn, Co, Fe, N, C, and O. Figure 3c,d,h,j illustrate the chemical states of these elements, specifically iron (Fe 2p) and zinc (Zn 2p) in Fe-ZIF-8, and cobalt (Co 2p) in Fe-ZIF-67. The presence of Zn(II) in Fe-ZIF-8 is indicated by the peaks at Zn 2p^1/2^ (1045 eV) and Zn 2p^3/2^ (1020 eV), observed in all samples. Similarly, the presence of Co(II) in Fe-ZIF-67 is confirmed by the peaks at Co 2p^1/2^ (796.22 eV) and Co 2p^3/2^ (780.7 eV). It is noteworthy that the carbon and nitrogen species on the surface of ZIF-8 and Fe-ZIF-67 are similar to those previously reported [28]. As expected, compared to ZIF-8 and ZIF-67, Fe-ZIF-8 and Fe-ZIF-67 show an additional Fe-Nx peak, indicating successful incorporation of Fe into the ZIF-8 and ZIF-67 frameworks using the IST strategy. The Zn 2p and Co 2p spectra both exhibit two primary components, 2p^3/2^ and 2p^1/2^, consistent with earlier findings [39]. Additionally, the spectral peaks of C and N shift towards lower binding energies, suggesting changes in the chemical interactions among C, N, Fe, and Zn atoms due to the addition of Fe and the activation process

**Figure 4:**
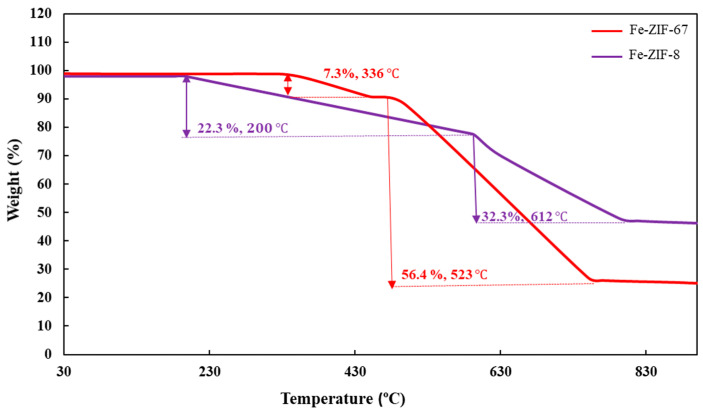
TGA pattern of Fe-ZIF-8 and Fe-ZIF-67.

The stability of Fe-ZIFs was evaluated by TGA, with particular emphasis placed on examining their chemical and thermal properties. The alterations in mass for both adsorbents are easily observed in Figure 4. The results for Fe-ZIF-8 showed two separate mass reductions observed at different temperature intervals. The initial decrease in mass, amounting to 22.3%, was recorded when the temperature reached 200 °C. Subsequently, a decline was observed at a temperature of 523 °C, leading to a proportional loss in mass of 56.4%. The plot of Fe-ZIF-67 exhibited two separate weight reductions occurring at specific temperatures: 336 °C, leading to a mass loss of 7.3%, and 612 °C, resulting in a significant mass loss of 32.3%. The weight reduction in Fe-ZIF-8 at a temperature of 200 °C was not ascribed to any identifiable structural degradation. Nevertheless, it is plausible to draw a correlation between this occurrence and the elimination of adsorbed water or unreacted 2-methylimidazole. On the other hand, prior research has provided evidence that the reduction in weight observed in synthesized ZIFs can be ascribed to the disintegration or deterioration of the underlying framework structure [43,44].

ICP-OES was employed to evaluate the iron concentration in samples subsequent to their exposure to a digestion procedure involving the use of a nitric acid (HNO_3_) solution, Table 1. The process of digestion facilitates the liberation of the desired metal ions from the zeolitic imidazolate framework structures, hence enabling their measurement and quantification. The investigation using ICP-OES yielded significant findings about the iron concentration in the samples. The research conducted demonstrated iron content values of 3.4 in both adsorbents. The quantitative measurements provide significant insights into the presence and concentration of iron in the various ZIF materials.

The Brunauer–Emmett–Teller method (BET) was used to evaluate the porosity characteristics of the synthesized ZIFs, including parameters such as pore volume, pore size, surface area, and Langmuir surface area, Table 2. The investigation produced noteworthy results regarding the porosity attributes of the materials. It is worth mentioning that an increase in the iron concentration inside the ZIF led to a decrease in the surface area and porosity properties of adsorbents. The presence of pollutants or other substances with the ability to induce structural alterations may be a potential explanation for the manifestation of this phenomena, leading to the closure or alteration of certain pores [45]. Furthermore, the porosity characteristics, including pore volume and pore size (as calculated by the Horvath–Kawazoe method), exhibited a comparable trend to that observed in the surface area. The decrease in the molar ratio of 2-methylimidazole (2-MIM) to zinc and cobalt led to a simultaneous fall in both the BET surface area and the total pore volume of the particles. The decline in performance can be ascribed to the detection of broader particle size distributions when the molar ratios of 2-MIM/Zn are decreased [46]. This observation presents empirical data that aligns with the concept that smaller particles have larger BET surface areas.

**Figure 5:**
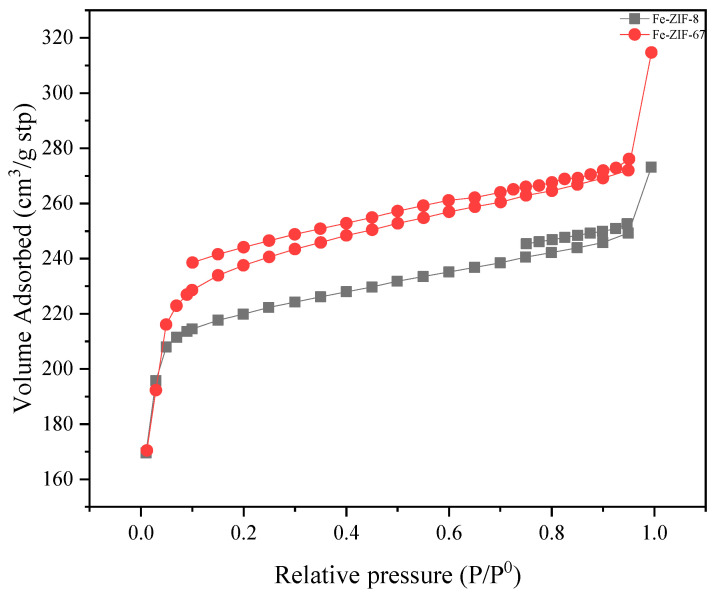
N_2_ adsorption/desorption patterns for Fe-ZIF-8 and Fe-ZIF-67.

The N_2_ adsorption method was utilized to assess the enduring permeability, microscopic porosity structure, and stability of the synthesized samples. Prior to N_2_ adsorption, all Fe ZIF samples were degassed under high vacuum (<10^−3^ mbar) at 150 °C for 12 h to remove adsorbed moisture and any labile guest species. After activation, samples were transferred under a dry N_2_ atmosphere to the adsorption port to avoid reabsorption of water. The nitrogen isotherm profile for the used absorbent materials can be categorized as Type I, as depicted in Figure 5. It is noteworthy that a hysteresis effect was identified in the N_2_ isotherm pattern exhibited by all samples. The observed phenomenon can be ascribed to the existence of variations in pore sizes, specifically the presence of mesopores (2–50 nm) that undergo capillary condensation. The behavior that has been observed is linked to the level of iron (Fe) incorporation into the framework structure. The occurrence of hysteresis events was observed in the structure of zeolitic imidazolate frameworks (ZIFs) with different levels of iron concentrations [47]. Furthermore, during the IST, the emergence of hierarchical materials can occur due to the liberation of gases resulting from chemical processes, such as the degradation of precursors. This phenomenon leads to the occurrence of hysteresis in the nitrogen isotherm. The isotherm profiles of the adsorbents, with iron loading of up to 5%, had minimal influence on the crystalline properties as a result of the iron exchange process.

**Figure 6:**
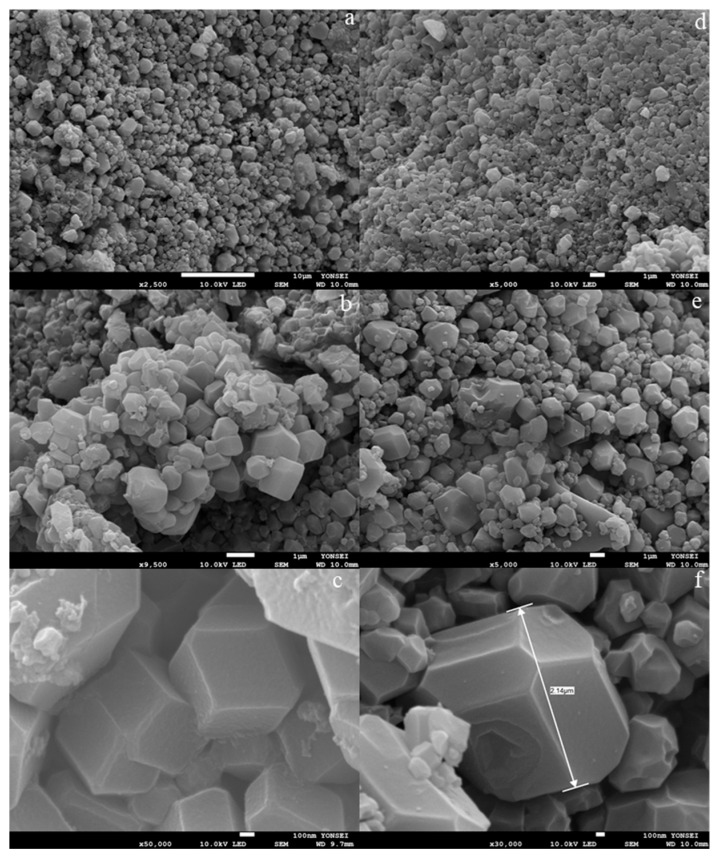
SEM pictures of Fe-ZIF-8 samples denoted as (**a**–**c**) and Fe-ZIF-67 samples denoted as (**d**–**f**) were acquired using the IST.

To enhance our comprehension of the physical characteristics, such as the shape and dispersion of particles within the specimens, SEM analysis was conducted and the results are presented in Figure 6. The morphology of Fe-ZIF-8 was seen to exhibit a rhombic dodecahedral structure, which is a characteristic feature of ZIF-8, as illustrated in Figure 6a,b. In a similar manner, it was observed that Fe-ZIF-67 consistently displayed a rhombic dodecahedral morphology, as depicted in Figure 6c,d. All the adsorbents exhibited notable iron dispersion and had a substantial degree of porosity. 

Furthermore, the use of scanning electron microscopy (SEM) images validates the high crystallinity and extensive distribution of nanoparticles. The likely reason for this result can be linked to the inadequate presence of a solvent in the IST technique, hence hindering the attainment of homogeneous materials. It is crucial to recognize that the creation of non-uniform particles by the IST technique is consistent with previous findings documented in the extant literature [48,49]. The results obtained from the field-emission scanning electron microscopy (FE-SEM) analysis revealed that the examined samples exhibited a range of particle sizes spanning from 250 nm to 800 nm, without any observable regularity or arrangement. The structural characteristics of Fe-ZIF-8 and Fe-ZIF-67 were found to exhibit rhombic dodecahedral and regular rhombic dodecahedral geometries, respectively. The utilization of energy dispersive spectrometry (EDS) mapping was employed as a means to examine the spatial characteristics and dispersion patterns of components. EDS maps (Figures S7 and S8) confirm uniform dispersion of Fe, Zn, Co, C, and N across the frameworks, matching expected stoichiometry and demonstrating successful in-situ incorporation of iron. This homogeneous distribution correlates with the high adsorption performance observed.

### 3.2. Adsorption Kinetics

Adsorption kinetics were fitted to pseudo-first-order and pseudo-second-order models (Figure S1). The pseudo-second-order model gave an excellent fit (R^2^ = 0.99), with rate constants and calculated q_e_ values matching experimental data (Table 3), indicating that chemisorption governs the process.

To directly assess the impact of Fe(III) incorporation on MG uptake, we performed parallel batch adsorption experiments on pure ZIF 8, Fe ZIF 8, pure ZIF 67, and Fe ZIF 67 under identical conditions. As shown in Table 4, pure ZIF 8 and ZIF 67 achieve q_max_ values of 310 ± 8 mg·g^−1^ and 345 ± 7 mg·g^−1^, respectively. The incorporation of Fe(III) increases q_max_ to 495 ± 3 mg·g^−1^ for Fe ZIF 8 and 552 ± 4 mg·g^−1^ for Fe ZIF 67, representing enhancements of 60% in both cases. These results unequivocally demonstrate that Fe doping significantly enhances MG adsorption capacity by introducing additional chemical binding sites, without altering the testing framework or conditions.

### 3.3. The Isotherm of Adsorption

Equilibrium isotherms measured at 25 °C, 35 °C, and 45 °C (Figures S2–S5) revealed an increase in adsorption capacity with temperature, confirming endothermic behavior. Freundlich parameters (Table 4) showed a rising K_F_ and 1/n < 1, consistent with favorable multilayer adsorption. As shown in Table 5, the Langmuir model for Fe-ZIF-8 and the Freundlich model for Fe-ZIF-67 display a higher correlation coefficient compared to other models, making them well-suited for determining the adsorption capacity. This indicates that monolayer adsorption occurred on the adsorbent, and the dye receptor groups were evenly distributed across the adsorbent surface. The Freundlich model’s n value of 1.35 suggests favorable uptake. In investigating multilayer surface adsorption, the Temkin model was applied, resulting in an isothermal correlation coefficient of 0.955 for Fe-ZIF-8 and 0.966 for Fe-ZIF-67, which does not support the existence of multilayer adsorption. The observed behaviors can be attributed to the requirement of a substantial amount of energy to promote the diffusion of large MG molecules into the pores of Fe-ZIF-8 and Fe-ZIF-67 [50]. Additionally, it was observed that the equilibrium adsorption capacity (q_e_) demonstrated a positive correlation with greater levels of the equilibrium concentration (C_e_). Subsequently, the data acquired from the isotherm experiments were subjected to analysis using four unique isothermal models, specifically the Langmuir model, the Freundlich model, the Temkin model, and the Dubinin–Radushkevich (D-R) model. Upon performing a comparative analysis, it was evident that the Freundlich model exhibited superior accuracy in determining parameters when compared to the Langmuir, Temkin, and D-R models. The results obtained from the experiment suggest that the adsorption of MG on synthesized ZIFs exhibits a multi-layer behavior. The discovery noted above suggests that the process of adsorption involves interactions taking place at several binding sites within the ZIF structures, highlighting the complex and varied nature of the adsorption mechanism for MG on these materials.

### 3.4. Thermodynamic Analysis

Table 6 displays the thermodynamic parameters that have been calculated for the adsorption of MG onto adsorbents. Figure S3 displays the computed free energy ΔG° at 298.15 K, 308.15 K, and 318.15 K, all registering positive values. This positivity signifies the non-spontaneity of MG adsorption on both adsorbents at the designated temperatures. With increasing temperature, the ΔG° values increase, emphasizing the heightened favorability of MG adsorption at elevated temperatures. The enthalpy (ΔH°) and entropy (ΔS°) of the adsorption process were determined from the slope and intercept of this plot. The calculated values for ΔH° and ΔS° stand at 6.72 kJ mol^−1^ and 4.145 kJ mol^−1^ K^−1^ for Fe-ZIF-8 and 15.69 kJ mol^−1^ and 36.07 kJ mol^−1^ K^−1^ for Fe-ZIF-67, respectively. Confirming the positive ΔH°, the MG adsorption onto ZIF-67 is established as an endothermic reaction. Additionally, the positive ΔS° suggests the desorption of several pre-adsorbed water molecules during MG adsorption onto ZIF-67, consistent with prior studies [51]. These results harmonize with the conclusions drawn from the analyses of kinetics and adsorption isotherms, collectively indicating that MG adsorption onto Fe-ZIF-8 and Fe-ZIF-67 is not only endothermic but also significantly influenced by chemical interactions.

### 3.5. Effect of Parameters on MG Adsorption

We systematically varied pH (3–11), adsorbent dosage (5–30 mg), initial MG concentration (20–120 mg·L^−1^), and temperature (298–318 K) (Figure S6). Optimal removal (>95%) was achieved at pH 6, 17.5 mg dosage, and 80 mg·L^−1^ MG; efficiency dropped under highly acidic or alkaline conditions due to changes in surface charge and speciation.

#### 3.5.1. Effect of pH Value on Adsorption Capacity

**Figure 7:**
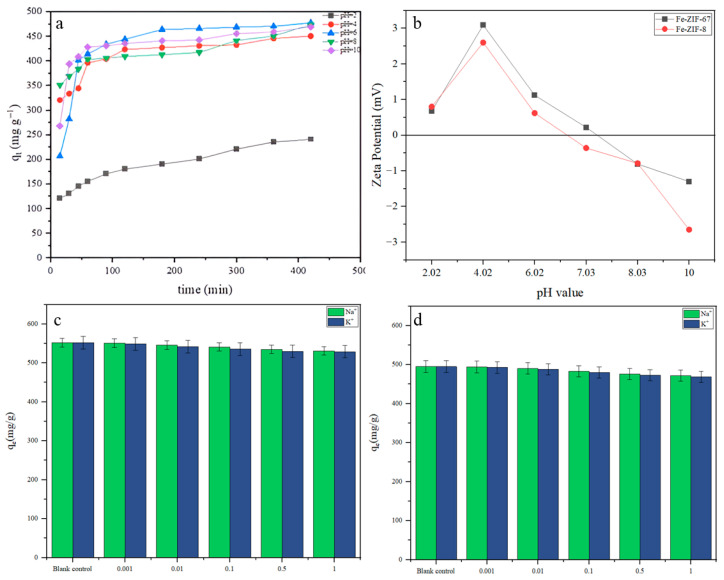
Effects of the pH (**a**), Zeta potential (**b**), and co-existing ions on the MG adsorption by (**c**) Fe-ZIF-67 and (**d**) Fe-ZIF-8.

For this experiment, 17.5 mg of the adsorbent was introduced into a solution containing 100 mL of an MG solution with a concentration of 80 mg·L^−1^. The mixture was left undisturbed for roughly seven hours to achieve adsorption equilibrium. The pH of the solution was modified by adding hydrochloric acid and sodium hydroxide solutions to attain pH values of 2.0, 4.0, 6.0, 7.0, 8.0, and 10.0 as determined by a pH meter (IP54, Mettler Toledo). Next, the adsorption characteristics for MG of the as-synthesized samples are characterized at various adsorbent pH levels using the aforementioned approach (Figure 7a). As shown in Figure 7a, the adsorption capacity of both Fe-ZIF-8 and Fe-ZIF-67 for MG increases sharply as the solution pH is raised from 2.0 to around 6.0–7.0, reaches a maximum near neutral pH, and then levels off or slightly declines at pH > 8.0. At strongly acidic pH (≤4), the ZIF surface is protonated (pH < pH_0_), carrying a net positive charge that repels the cationic MG molecules, leading to poor uptake. As the pH approaches the point of zero charge (pH_0_ ≈ 6–7, see Section 3.5.2), surface deprotonation generates negative sites that electrostatically attract MG, causing the steep rise in q_e_. Above pH 8, although the surface remains negatively charged and continues to favor MG binding, two effects moderate further gains: (1) the onset of MG hydrolysis/polymerization at very high pH, and (2) competition with OH^−^ for active sites, both of which slightly diminish the net increase in q_e_. The plateau in adsorption beyond neutral pH thus reflects a balance between enhanced electrostatic attraction and these secondary phenomena. Overall, the optimum pH range (6–8) aligns well with typical wastewater conditions, underscoring the practical appeal of these Fe-modified ZIFs for MG removal.

#### 3.5.2. Zero Charge Point pH_0_ of the Adsorbents

As shown in Figure 7b, the point of zero charge (pH_0_) was determined using the solid addition method to analyze the surface electrical characteristics of the adsorbents. In summary, the pH of the solution was modified from 2.0 to 10.0 by employing hydrochloric acid and sodium hydroxide solutions. Subsequently, 17.5 mg of the adsorbent was introduced into 5 mL of 0.1 M KCl solutions, each with different starting pH (pH_i_) values. Subsequently, the mixes were agitated at ambient temperature for a duration of 24 h. The pH meter was used to measure the final pH (pH_f_). A graph was created by plotting the difference in pH (pH_i_–pH_f_) against the initial pH (pH_i_). The pH value at which the difference in pH becomes zero was determined as the point of zero charge (pH_0_).

#### 3.5.3. Effect of Co-Existing Ions

MG in adsorbents can be affected by the presence of other ions found in natural water, as these ions can compete for adsorption on the accessible sites. In order to replicate the intricate conditions of real wastewater, varying quantities of K^+^ and Na^+^ ions were introduced to evaluate their impact on MG adsorption. Figure 7c and Figure 6d demonstrate that the presence of Na^+^ and K^+^ ions had a comparable effect on the adsorption of MG. Modestly elevating the concentration of these disruptive cations had only a minimal impact on MG adsorption. The presence of coexisting ions did not have a significant impact on the adsorption capacity for MG, suggesting that adsorbents have excellent selectivity for MG.

### 3.6. Adsorption Mechanism

Through a thorough examination of kinetics, adsorption isotherms, and thermodynamic data, it has been determined that the adsorption of MG is predominantly governed by a chemical interaction with adsorbents (Table 5 and Table 6). Importantly, the pH of the solution did not indicate that electrostatic forces were the main driving factor behind the adsorption of MG. This suggests that other chemical interactions, particularly in the case of ZIF-67, are significantly involved in the adsorption process. As illustrated in Figure 6, both ZIFs possess a rhombic dodecahedral architecture consisting of 2-MIM moieties linked to Zn^2+^, Co^2+^, and Fe^3+^ cations. The molecular structure of 2-MIM includes an imidazole ring that possesses two double bonds and a pair of electrons originating from the protonated nitrogen. These structural features collectively contribute to the interactions occurring on the planar surface of the imidazole ring. It is widely accepted in scientific literature that the imidazole ring has aromatic properties, allowing it to participate in π–π stacking interactions with other aromatic compounds, such as MG. The π–π stacking interaction is considered a crucial element that increases the adsorption capacity of ZIFs for MG, resulting in their exceptional ability to remove this dye from water solutions.

**Figure 8:**
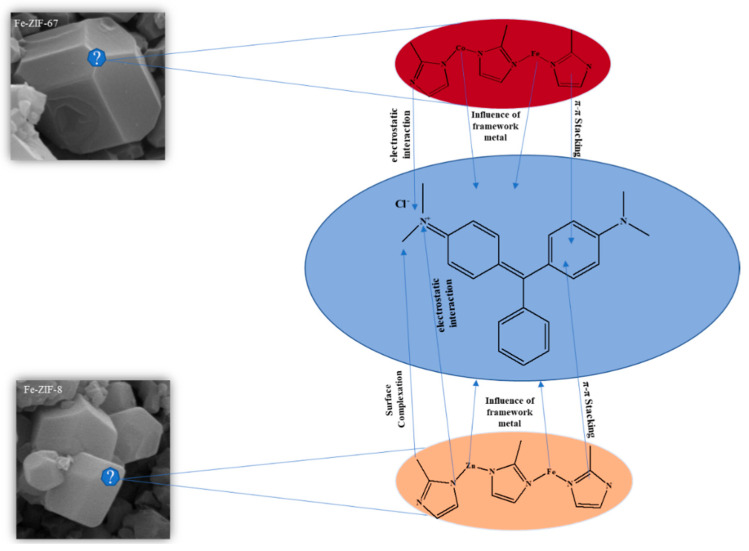
Mechanism proposed for the adsorption of MG over Fe-ZIF-8 and Fe-ZIF-67.

In addition, the nitrogen atom in the imidazole ring can create hydrogen bonds with the MG molecules, namely between the protonated nitrogen in MG and the lone pair of electrons on the nitrogen atoms of the imidazole. The heightened adsorption efficiency found is likely due to the presence of both π–π stacking and hydrogen bonding in this dual interaction mechanism. Moreover, the thermodynamic characteristics, notably the endothermic adsorption enthalpy and positive adsorption entropy, suggest that the main factor driving the adsorption of MG onto the ZIFs is entropy (Table 6). This indicates that the adsorption process is primarily controlled by the disturbance of the hydrophobic hydration shell that surrounds MG molecules in water-based settings. Within this particular setting, MG molecules are commonly encased by an organized configuration of water molecules, bearing a resemblance to clathrate-like formations. The adsorption process is likely to disturb the organized water network, making it easier for MG to be adsorbed onto the ZIF surfaces (Figure 8).

### 3.7. Reusability of Adsorbent

**Figure 9:**
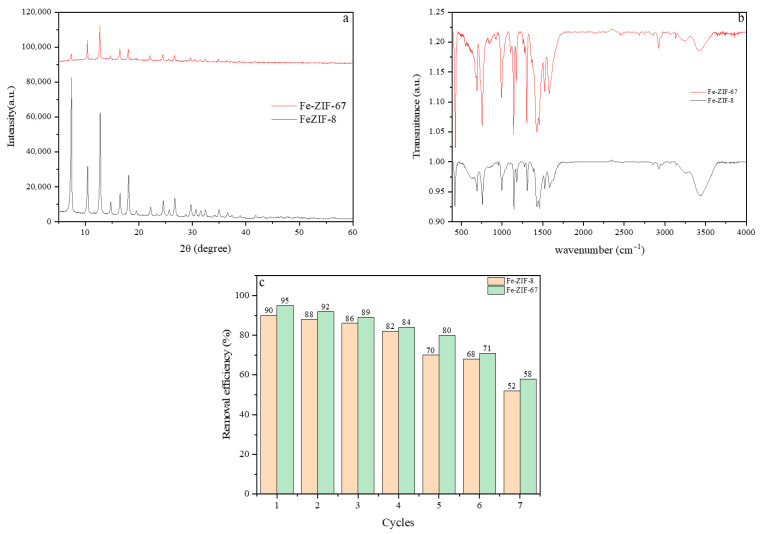
(**a**) XRD spectrum, (**b**) FT-IR patterns, and (**c**) removal efficiency of Fe-ZIF-8, Fe-ZIF-67 adsorbents after seven cycles.

The ability to reuse the adsorbent is of paramount importance in practical applications, as it can significantly contribute to cost reduction. To evaluate the reusability of samples, recycling experiments were performed over seven consecutive adsorption–desorption cycles. In the desorption process, Fe-ZIF-8 and Fe-ZIF-67, loaded with MG, were thoroughly washed with dichloromethane under soaking conditions. As illustrated in Figure 9, there was only a negligible reduction in the removal efficiency of Fe-ZIF-8 and Fe-ZIF-67 with each successive adsorption cycle. This result indicates that these materials can be effectively and repeatedly used without a significant decline in their adsorption performance. Even after three cycles, Fe-ZIF-8 and Fe-ZIF-67 still maintained a high removal efficiency of 86% and 89% for Fe-ZIF-8 and Fe-ZIF-67, demonstrating their excellent reusability. Also, XRD and FTIR analyses were conducted to measure the physical and chemical stability of ZIFs after batch experiments.

## 4. Conclusions

In this study, Fe-ZIF-8 and Fe-ZIF-67 were successfully synthesized via an environmentally friendly in situ thermal (IST) method without the use of solvents or additives. This scalable and cost-effective approach enabled the rapid production of highly porous frameworks, with 2-methylimidazole serving as ligand, solvent, and hydrophilic agent. The synthesized materials required no post-synthesis activation and exhibited well-defined crystallinity. The Fe-ZIFs demonstrated exceptional adsorption capacities for malachite green (495 mg g^−1^ for Fe-ZIF-8 and 552 mg g^−1^ for Fe-ZIF-67), outperforming previously reported adsorbents. Adsorption behavior followed the pseudo-second-order kinetic model and was well described by the Freundlich isotherm, indicating multilayer adsorption. Enhanced removal under acidic conditions was attributed to Fe acting as an electron acceptor, while alkaline conditions led to passivation by iron oxide/hydroxide layers. The high performance was due to the mesoporous structure and multiple interaction mechanisms, including electrostatic forces, hydrogen bonding, π–π stacking, and surface complexation. The adsorbents also showed good reusability over multiple cycles. Overall, the IST method offers a green, efficient, and scalable strategy for producing high-quality ZIF materials, contributing significantly to sustainable adsorbent development for water purification.

## Figures and Tables

**Table 1:** Metal content in Fe-ZIF-8 and Fe-ZIF-67 as determined by ICP-OES after acid digestion. RSD refers to the relative standard deviation over three replicate measurements.

Samples	Co (%wt)	Fe (%wt)	Zn (%wt)	RSD (%)
Fe-ZIF-8	-	3.4	36.4	0.2815
Fe-ZIF-67	36.5	3.5	-	0.2324

**Table 2:** The objective of this study is to investigate and compare the surface area and porosity characteristics of the adsorbent samples. Samples were activated at 150 °C under <10^−3^ mbar vacuum for 12 h. Total pore volume (V_t_) was calculated from the adsorption branch at p/p_0_ = 0.95.

Sample	BET (m^2^ g^−1^)	Langmuir (m^2^ g^−1^)	Pore Size (nm)	V_t_ (cm^3^·g^−1^, at p/p_0_ = 0.95)
Fe-ZIF-8	855.5 ± 0.1	967.8	1.975	0.42 ± 0.01
Fe-ZIF-67	927.6 ± 0.1	1012.1	2.099	0.49 ± 0.01

**Table 3:** The estimated kinetic parameters for the pseudo-first-order and pseudo-second-order reduction models, as well as the Intraparticle diffusion model.

Sample	$q_{e,exp}$ (mg g^−1^)	Pseudo-First-Order Kinetic Model			Pseudo-Second-Order Kinetic Model			Intraparticle Diffusion Model		
		$q_{e,cal}$ (mg g^−1^)	$k_{1}$ (min^−1^)	$R^{2}$	$q_{e,cal}$ (mg g^−1^)	$k_{2}$ (g mg^−1^ min^−1^)	$R^{2}$	$k_{diff}$ (mg g^−1^ min^−0.5^)	c	$R^{2}$
Fe-ZIF-67	65.0 ± 0.5	34.1 ± 0.2	0.00004	0.98	63.2 ± 0.4	0.0012	0.99	0.252	265.31 ± 0.3	0.988
Fe-ZIF-8	75.0 ± 0.5	66.2 ± 0.1	0.00005	0.86	74.1 ± 0.5	0.0015	0.99	0.204	235.88 ± 0.2	0.975

**Table 4:** Maximum adsorption capacities for MG on pure and Fe-doped ZIFs measured under identical batch adsorption conditions.

Adsorbent	q_max_ (mg·g^−1^)
Pure ZIF-8	310 ± 8
Fe-ZIF-8	495 ± 3
Pure ZIF-67	345 ± 7
Fe-ZIF-67	552 ± 4

**Table 5:** Model parameters for MG removal according to the best-fitting Langmuir–Freundlich–Temkin, D-R isotherm systems.

Samples	T (K)	Langmuir Isotherm			Freundlich Isotherm			
		q_m_ (mg g^−1^)	K_L_ (L mg^−1^)	R^2^	K_f_ (mg^(1–−1/n)^ L^(1/n)^ ^g−1^)	n	R^2^	
Fe-ZIF-67	298.15	454.5 ± 0.7	0.10	0.985	32.0 ± 0.4	1.352	0.992	
	308.15	476.1 ± 0.8	0.17	0.986	43.3 ± 0.5	1.354	0.981	
	318.15	666.6 ± 0.6	0.18	0.964	53.9 ± 0.4	1.274	0.983	
Fe-ZIF-8	298.15	625.0 ± 0.7	0.04	0.998	28.7 ± 0.5	1.309	0.993	
	308.15	796.2 ± 0.6	0.04	0.998	38.6 ± 0.4	1.308	0.989	
	318.15	1428.5 ± 0.8	0.03	0.964	52.9 ± 0.4	1.251	0.999	
Samples	T (K)	Temkin isotherm			D-R isotherm model			
		B_T_ (J mol^−1^)	K_T_ (L g^−1^)	R^2^	q_m_ (mg g^−1^)	β(mol^2^ kJ^−2^)	E (kJ mol^−1^)	R^2^
Fe-ZIF-67	298.15	104.1 ± 0.3	0.74	0.966	240.3 ± 0.7	1.41 × 10^−6^	595.8	0.974
	308.15	119.9 ± 0.4	0.71	0.993	296.1 ± 0.5	1.06 × 10^−6^	687.0	0.962
	318.15	164.9 ± 0.2	1.06	0.998	456.8 ± 0.6	9.99 × 10^−7^	707.4	0.969
Fe-ZIF-8	298.15	101.8 ± 0.5	0.71	0.960	233.0 ± 0.8	1.45 × 10^−6^	587.9	0.985
	308.15	123.1 ± 0.3	0.85	0.955	269.5 ± 0.6	9.43 × 10^−7^	728.1	0.945
	318.15	176.4 ± 0.2	0.98	0.987	461.1 ± 0.7	9.28 × 10^−6^	734.1	0.932

**Table 6:** Disposal of MG as a function of thermodynamics.

Samples	T (K)	∆G(KJ/mol)	∆H(KJ/mol)	∆S(JK·mol)	R^2^
Fe-ZIF-67	298.15	4994.656	15.691	36.075	0.984
	308.15	4443.977			
	318.15	4281.540			
Fe-ZIF-8	298.15	7960.350	6.720	4.145	0.982
	308.15	7986.499			
	318.15	8043.925			

## Data Availability

Data are contained within the article and Supplementary Materials.

## Supplementary Materials

The following supporting information can be downloaded at: https://www.mdpi.com/article/10.3390/nano15141097/s1, Figure S1. Kinetic models for the removal of MG by ZIFs. Figure S2. Langmuir isotherm model for the removal of MG by Fe-ZIFs. Figure S3. Freundlich isotherm model for the removal of MG by Fe-ZIFs. Figure S4. The Temkin isotherm model for the removal of MG by Fe-ZIFs. Figure S5. D-R isotherm model for the removal of MG by Fe-ZIFs. Figure S6. Adsorption capacity of Fe-ZIF-8 under different conditions: (a) varying adsorbent doses and (b) varying MG concentrations. Adsorption capacity of Fe-ZIF-67 under different conditions: (c) varying adsorbent doses and (d) varying MG concentration. Figure S7. Energy dispersive spectrometry (EDS) mapping of Ni-ZIF-8 obtained by the IST approach. Figure S8. Energy dispersive spectrometry (EDS) mapping of Ni-ZIF-8 obtained by the IST approach. Figure S9. The color change with different percentages of Fe (a) ZIF-8, (b) Fe_5%_ZIF-8, (c) Fe_10%_ZIF-8, (d) ZIF-67, (e) Fe_5%_ZIF-67, and (f) Fe_10%_ZIF-67.
