# Supplementary material for: Engineering Fe-Modified Zeolitic Imidazolate Frameworks (Fe-ZIF-8 and Fe-ZIF-67) via In Situ Thermal Synthesis for Enhanced Adsorption of Malachite Green from Aqueous Solutions: A Comprehensive Study of Isotherms, Kinetics, and Thermodynamics
Source: Nanomaterials (Basel). 2025 Jul 15;15(14):1097. doi: 10.3390/nano15141097 (PMC12298564; doi:10.3390/nano15141097)
Supplement: Supplementary file 1 [file nanomaterials-15-01097-s001.zip › nanomaterials-3742723-supplementary.pdf]

Article

Engineering Fe-Modified Zeolitic Imidazolate Frameworks (Fe-ZIF-8 and Fe-ZIF-67) via In-situ Thermal Synthesis for Enhanced Adsorption of Malachite Green from Aqueous Solutions: A Comprehensive Study of Isotherms, Kinetics, and Thermodynamics

Alireza Pourvahabi Anbari [1,2], Shima Rahmdel Delcheh [4], Muhammad Kashif [2,5], Alireza Ranjbari [2,5], Mohammad Karbalaei Akbari [2,3], Serge Zhuiykov [2,3], Philippe M. Heynderickx [2,5], Francis Verpoort [4*]

[1] Center for Green Chemistry and Environmental Biotechnology, Ghent University Global Campus, Incheon, 406-840 South Korea; Alireza.Pourvahabi@ghent.ac.kr (A.P.A); m.kashif@ghent.ac.kr (M.K.); alireza.ranjbari@ugent.be (A.R.); mohammad.akbari@ugent.be (M.K.A.); serge.zhuiykov@ghent.ac.kr (S.Z.); philippe.heynderickx@ghent.ac.kr (P.M.H.)

[2] State Key Laboratory of Advanced Technology for Materials Synthesis and Processing, Wuhan University of Technology, 430070 Wuhan, P.R. China; shima.rahmdel@yahoo.com (S.R.D.); francis@whut.edu.cn (F.V.)

[3] Research School of Chemical and Biomedical Technologies, National Research Tomsk Polytechnic University, Lenin Avenue 30, Tomsk 634050, Russia

[4] Joint Institute of Chemical Research (FFMiEN), Peoples Friendship University of Russia (RUDN University), 117198 Moscow, Russia.

[5] Department of Chemistry, Faculty of Science, Ghent University, B-9000 Gent, Belgium

[6] Department of Green Chemistry and Technology, Faculty of Bioscience Engineering, Ghent University, Ghent, B-9000, Belgium

[7] Department of Solid-State Sciences, Faculty of Science, Ghent University, 9000 Ghent, Belgium

*Correspondence: francis@whut.edu.cn

Contents

Figure S1. Kinetic Models for removal MG by ZIFs…………………………………………………………………..3

Figure S2. Langmuir isotherm model for removal of MG by Fe-ZIFs……………………………………………….....4

Figure S3. Freundlich isotherm model for removal of MG by Fe-ZIFs ………………………………………..…….4

Figure S4. Temkin isotherm model for removal of MG by Fe-ZIFs …………………………………………..…….5

Figure S5. D-R isotherm model for removal of MG by Fe-ZIFs ………………….…………………………..…….5

Figure S6. Adsorption capacity of Fe-ZIF-8 under different conditions: a) varying adsorbent doses and b) varying MG concentrations. Adsorption capacity of Fe-ZIF-67 under different conditions: c) varying adsorbent doses and d) varying MG concentrations……………………………………………………………………………………...…….6

Figure S7. Energy dispersive spectrometry (EDS) mapping of Ni-ZIF-8 obtained by the IST approach…..…..……7

Figure S8. Energy dispersive spectrometry (EDS) mapping of Ni-ZIF-8 obtained by the IST approach….…………8

Figure S9. The color change with different percentage of Fe (a) ZIF-8 (b) $Fe_{5\%}$ZIF-8 (c) $Fe_{10\%}$ZIF-8 (d) ZIF-67 (e) $Fe_{5\%}$ZIF-67 (f) $Fe_{10\%}$ZIF-67 ……………………………………………………………………………………………….9

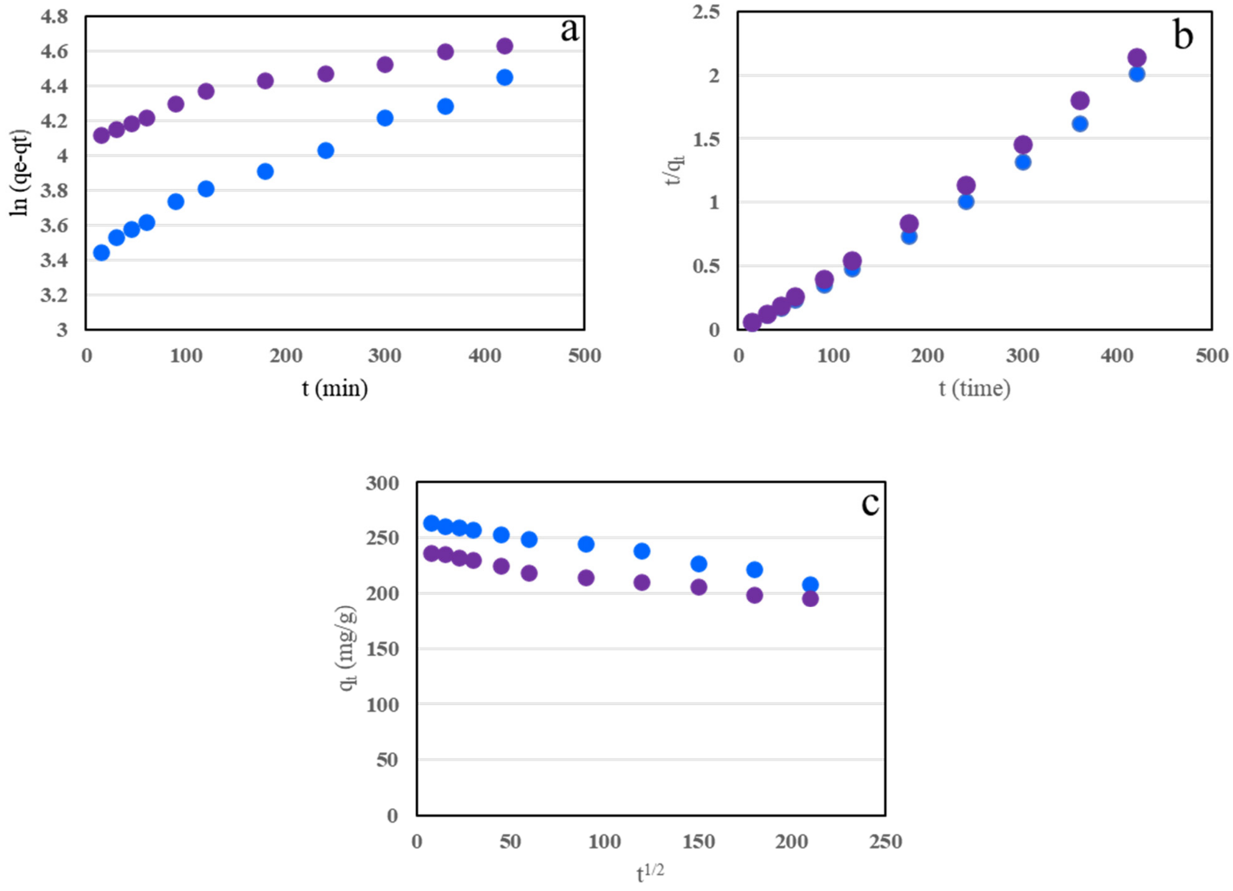

Figure S1. (a) pseudo-first-order (b) pseudo-second-order models and (c) Intraparticle diffusion models for kinetic data for removal of MG by Fe-ZIF-8 (purple) and Fe-ZIF-67 (blue).

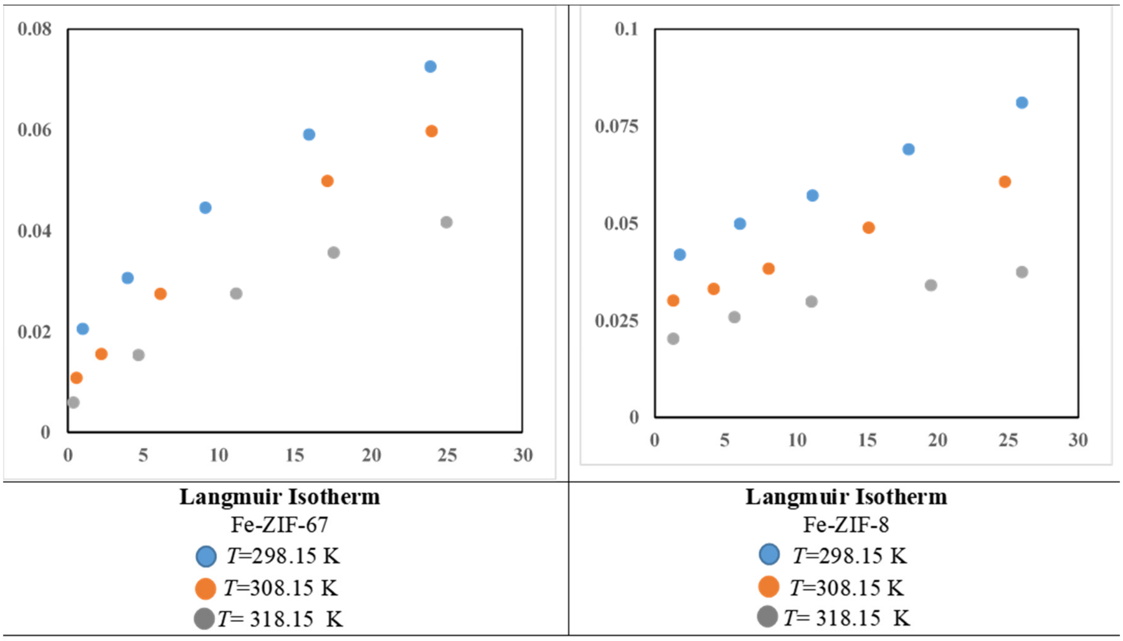

Figure S2. Langmuir isotherm model for removal of MG by Fe-ZIF-8 and Fe-ZIF-67.

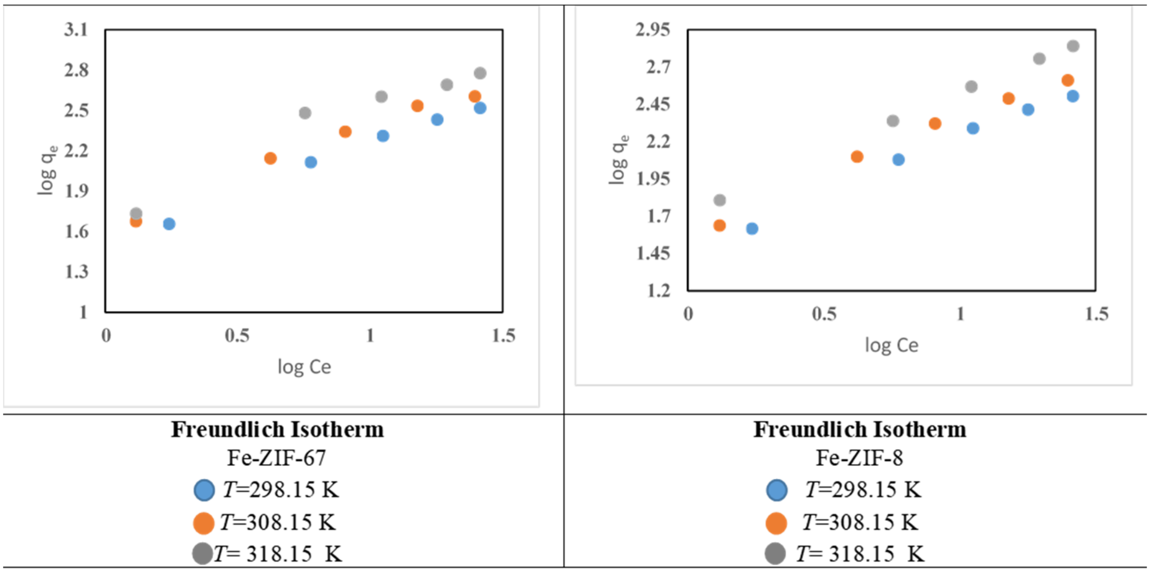

Figure S3. Freundlich isotherm model for removal of MG by Fe-ZIF-8 and Fe-ZIF-67.

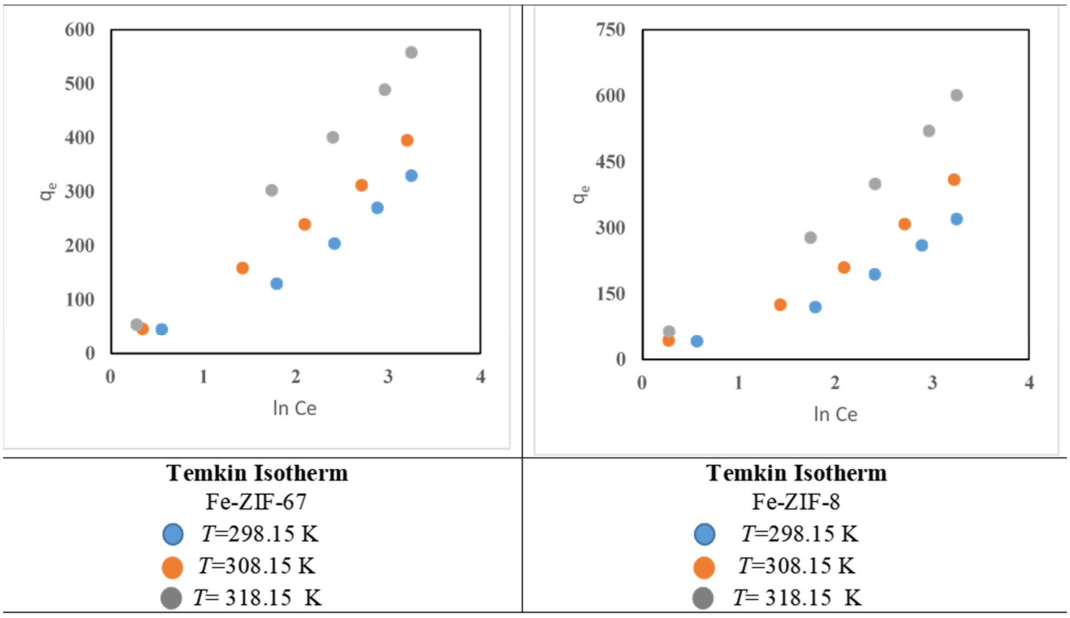

Figure S4. Temkin isotherm model for removal of MG by Fe-ZIF-8 and Fe-ZIF-67.

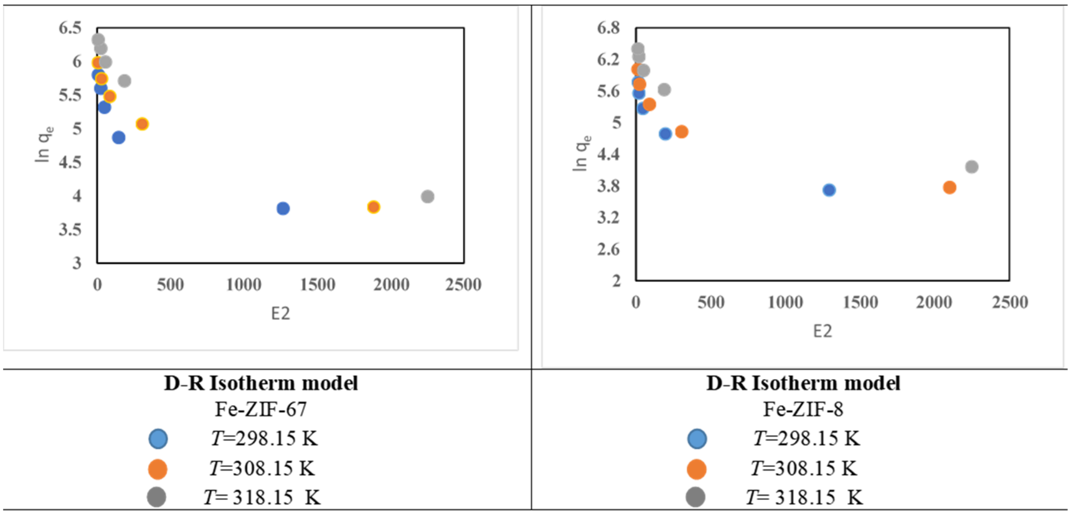

Figure S5. D-R isotherm model for removal of MG by Fe-ZIF-8 and Fe-ZIF-67.

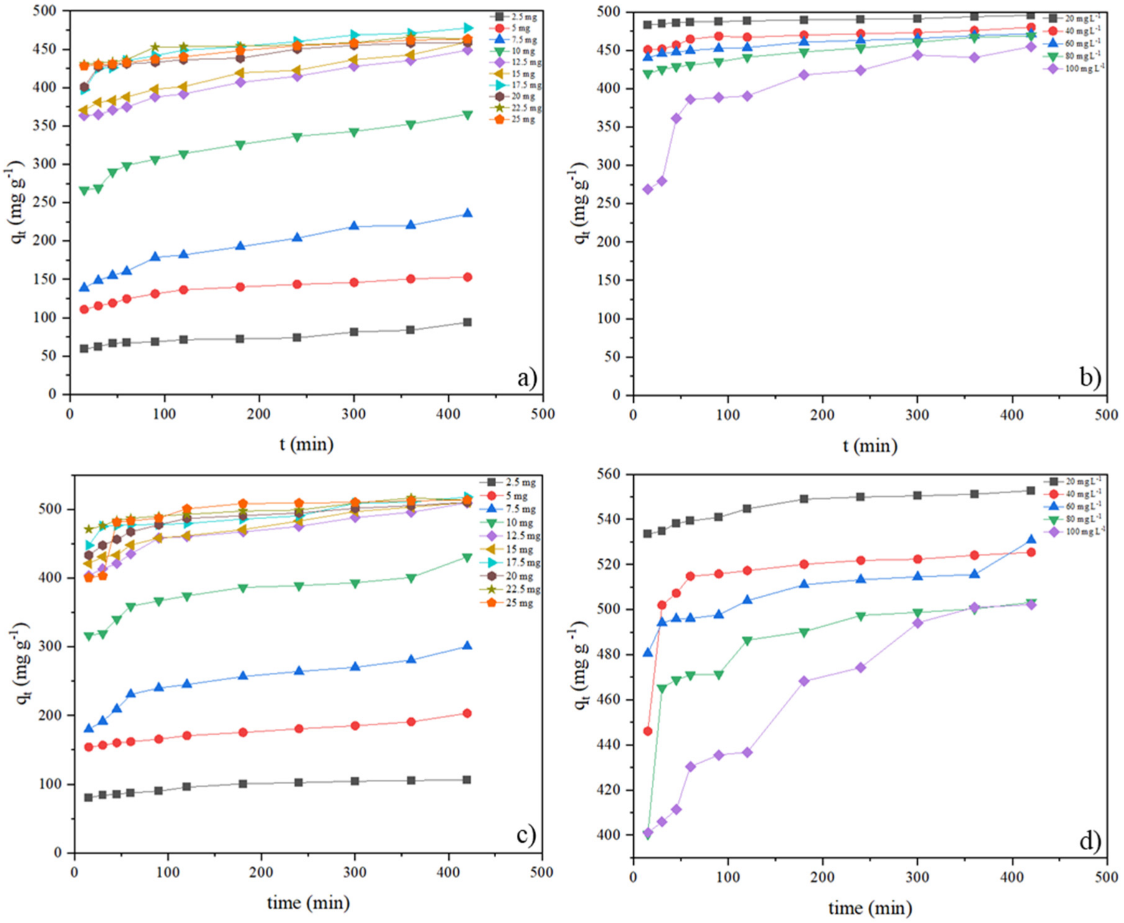

Figure S6. Adsorption capacity of Fe-ZIF-8 under different conditions: a) varying adsorbent doses and b) varying MG concentrations. Adsorption capacity of Fe-ZIF-67 under different conditions: c) varying adsorbent doses and d) varying MG concentrations.

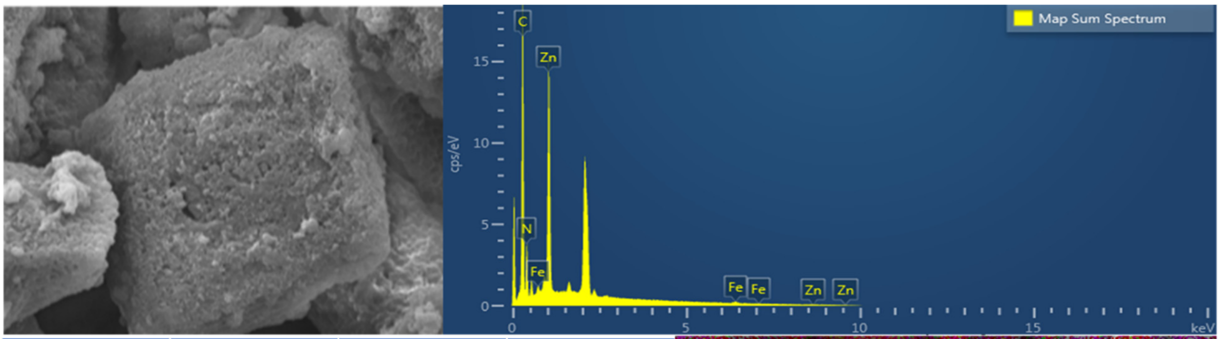

Element	Wt%	Wt% Sigma	Atomic %
C	48.55	0.37	64.27
N	25.92	0.40	29.41
Fe	2.63	0.37	0.75
Zn	22.90	0.21	5.57
Total:	100.00		100.00

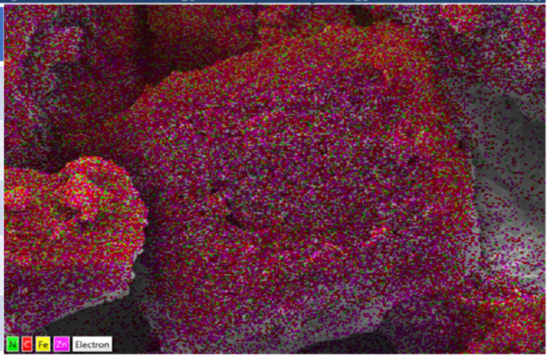

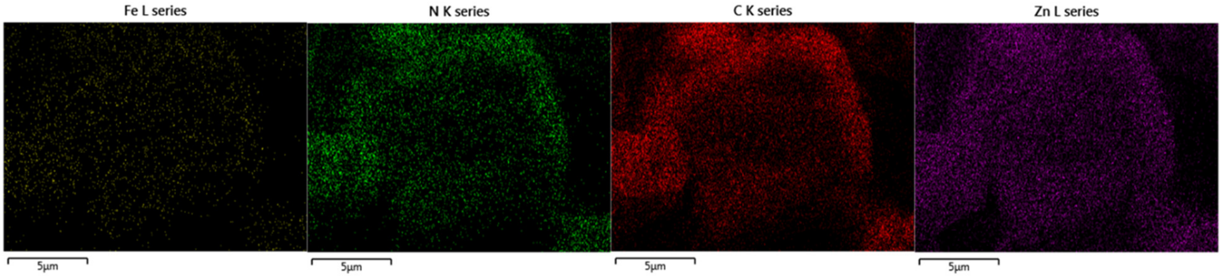

Figure S7. Energy dispersive spectrometry (EDS) mapping of Fe-ZIF-8 obtained by the IST approach

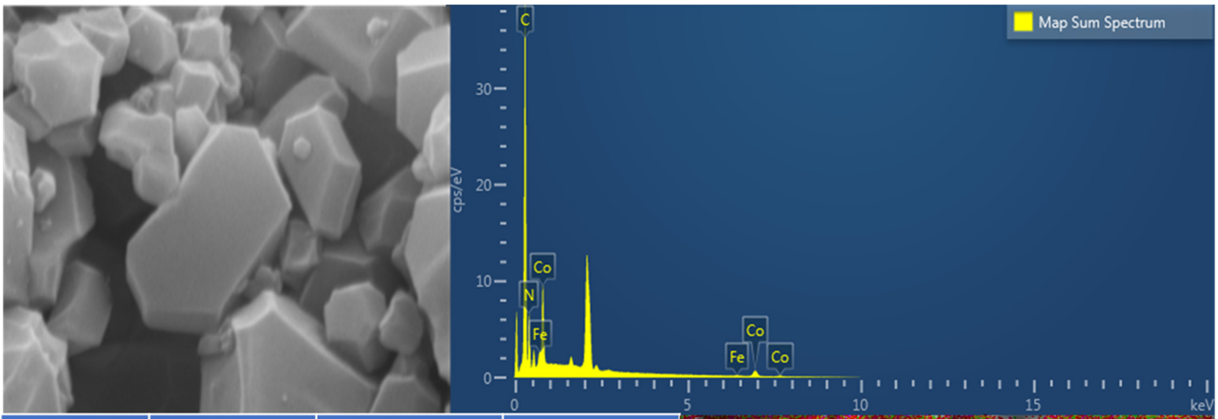

Element	Wt%	Wt% Sigma	Atomic %
C	51.02	0.33	64.57
N	27.53	0.34	29.88
Fe	0.99	0.26	0.27
Co	20.46	0.28	5.28
Total:	100.00		100.00

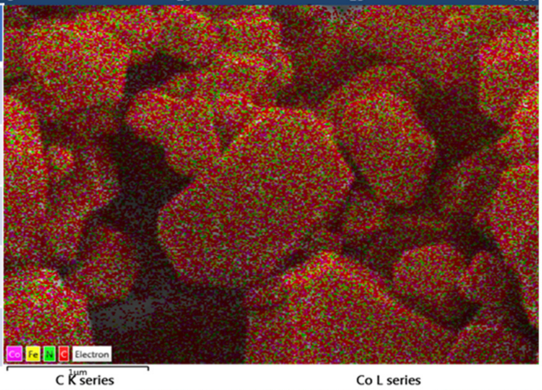

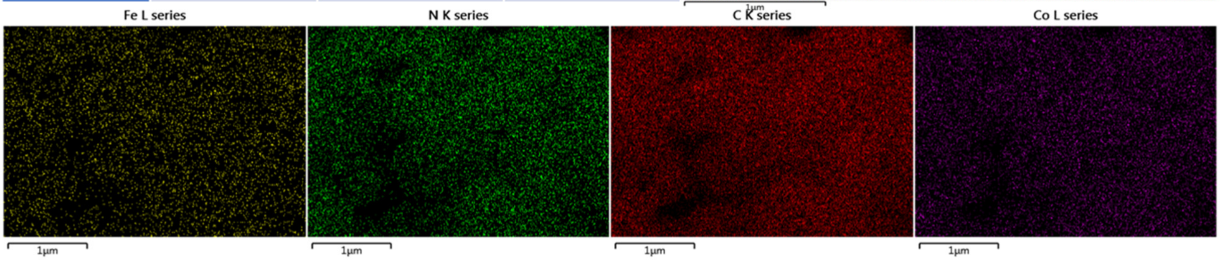

Figure S8. Energy dispersive spectrometry (EDS) mapping of Fe-ZIF-67 obtained by the IST approach

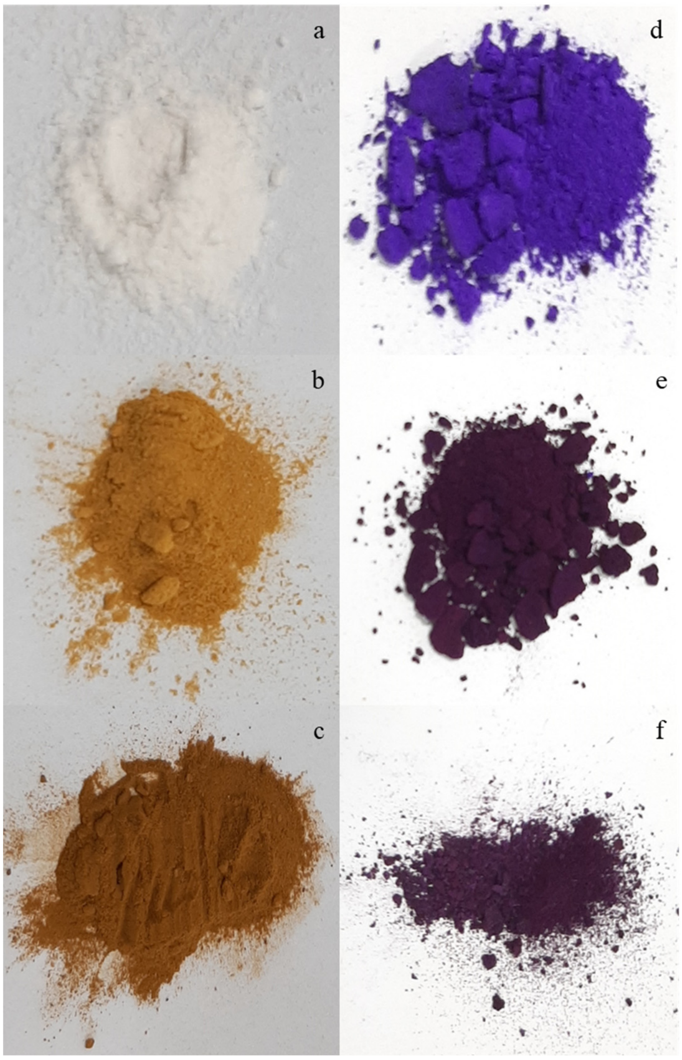

Figure S9. The color change with different percentage of Fe (a) ZIF-8 (b) $Fe_{5\%}$ZIF-8 (c) $Fe_{10\%}$ZIF-8 (d) ZIF-67 (e) $Fe_{5\%}$ZIF-67 (f) $Fe_{10\%}$ZIF-67
